# Microdialysis Monitoring of CSF Parameters in Severe Traumatic Brain Injury Patients: A Novel Approach

**DOI:** 10.3389/fneur.2014.00159

**Published:** 2014-09-02

**Authors:** Eric P. Thelin, David W. Nelson, Per Hamid Ghatan, Bo-Michael Bellander

**Affiliations:** ^1^Section for Neurosurgery, Department of Clinical Neuroscience, Karolinska Institutet, Karolinska University Hospital Solna, Stockholm, Sweden; ^2^Section of Anesthesiology and Intensive Care, Department of Physiology and Pharmacology, Karolinska Institutet, Stockholm, Sweden; ^3^Department of Clinical Neuroscience, Karolinska Institutet, Stockholm, Sweden

**Keywords:** TBI, microdialysis, cerebrospinal fluid, lactate, pyruvate, outcome

## Abstract

**Background:** Neuro-intensive care following traumatic brain injury (TBI) is focused on preventing secondary insults that may lead to irreversible brain damage. Microdialysis (MD) is used to detect deranged cerebral metabolism. The clinical usefulness of the MD is dependent on the regional localization of the MD catheter. The aim of this study was to analyze a new method of continuous cerebrospinal fluid (CSF) monitoring using the MD technique. The method was validated using conventional laboratory analysis of CSF samples. MD-CSF and regional MD-Brain samples were correlated to patient outcome.

**Materials and Methods:** A total of 14 patients suffering from severe TBI were analyzed. They were monitored using (1) a MD catheter (CMA64-iView, *n* = 7448 MD samples) located in a CSF-pump connected to the ventricular drain and (2) an intraparenchymal MD catheter (CMA70, *n* = 8358 MD samples). CSF-lactate and CSF-glucose levels were monitored and were compared to MD-CSF samples. MD-CSF and MD-Brain parameters were correlated to favorable (Glasgow Outcome Score extended, GOSe 6–8) and unfavorable (GOSe 1–5) outcome.

**Results:** Levels of glucose and lactate acquired with the CSF-MD technique could be correlated to conventional levels. The median MD recovery using the CMA64 catheter in CSF was 0.98 and 0.97 for glucose and lactate, respectively. Median MD-CSF (CMA 64) lactate (*p* = 0.0057) and pyruvate (*p* = 0.0011) levels were significantly lower in the favorable outcome group compared to the unfavorable group. No significant difference in outcome was found using the lactate:pyruvate ratio (LPR), or any of the regional MD-Brain monitoring in our analyzed cohort.

**Conclusion:** This new technique of global MD-CSF monitoring correlates with conventional CSF levels of glucose and lactate, and the MD recovery is higher than previously described. Increase in lactate and pyruvate, without any effect on the LPR, correlates to unfavorable outcome, perhaps related to the presence of erythrocytes in the CSF.

## Introduction

Traumatic brain injury (TBI) is a common cause of death and disability, increasing globally, with subsequent rising costs for society (1). The neuro-intensive care following TBI is focused on monitoring and preventing harmful secondary insults that may lead to irreversible brain damage (2, 3). The microdialysis (MD) technique is used to analyze focal brain biochemistry in patients suffering from TBI, examining concentrations of pyruvate, glucose, glycerol, and lactate in the extracellular fluid (ECF) (4). Glucose, being the main substrate for brain energy metabolism, will through glycolysis become pyruvate, which in normoxic conditions enters the mitochondria and becomes part of the citrate cycle. During hypoxic conditions, energy production will decrease while lactate levels, and the lactate:pyruvate ratio (LPR), will increase as a sign of tissue ischemia (5). In contrast, if pyruvate levels remain normal and lactate levels increase, ongoing mitochondrial dysfunction has been suggested (6). Glycerol, abundant in cell membrane, might be released and increase in the ECF as a sign of ongoing cell death (7).

Despite a consensus report on the clinical use of MD for TBI patients (8), the translation of the technique from research to bedside has been slow (9). An obstacle with the MD technique is the catheter placement. In order to optimize the monitoring capabilities, the catheter has been suggested to be best placed in the border zone of injuries, monitoring focal tissue at risk (8, 10–12). However, the results yielded by different catheter placements, and their correlation to outcome, have been questioned (13), and a pericontusional area is not readily detectable in diffuse TBI. Even if pericontusional tissue is monitored with MD, monitoring has been shown to present very heterogeneous metabolic results, proving accurate focal monitoring difficult (14). In contrast, a different approach to monitor potential variations has also been advocated, where the catheter is placed in non-pericontusional, non-affected brain tissue in order to detect more global metabolic changes (15).

The ECF and the cerebrospinal fluid (CSF) have been shown to demonstrate comparable pharmacokinetics and concentrations of administered drugs (16, 17). Also, data suggest that there is a flow of proteins between the ECF and CSF (18). Hence, analyzing the CSF could reflect changes in the ECF. Studies on the flow of metabolites, such as glucose, lactate, pyruvate, and glycerol between ECF and CSF are however limited. In contrast to proteins, their movements are often facilitated by their small size yet regulated by transporter proteins commonly following the concentration gradient between different compartments.

A MD-sampling of CSF is motivated to avoid infectious complications. About 10% of neurosurgical cases suffer from some kind of surgical-related infection, with about 4% risk of developing bacterial meningitis (BM) (19). Decreased CSF-glucose and increased CSF-lactate levels are since long recognized changes in BM (20), but also important predictors for developing a clinical BM following neurosurgical procedures (21). This study presents a CSF monitoring method using the MD technique. One of the major benefits of the current monitoring setup is that it is in a closed system, lowering the risks of infection and other mechanical complications.

### Aims

The aim was to validate a method of global MD monitoring by using a MD catheter placed in CSF. As a secondary aim, conventional CSF samples were used to calculate the recovery of the MD catheter and to correlate MD-CSF and MD-Brain parameters to patient outcome.

## Materials and Methods

### Inclusion criteria

Fourteen patients suffering from severe TBI were included between January 1, 2010 and December 31, 2012. The slow inclusion rate was due to the intermittent availability of the first author and did not represent a patient-selection process. The patients were monitored with an intracerebral 20 kDa MD catheter (MD-Brain). An additional 20 kDa MD catheter (CMA 64 IView, CMA Microdialysis AB, Solna, Sweden) was placed in the CSF through a LiquoGuard^®^ pump system (Möller-Medical, Fulda, Germany) (MD-CSF). The study was approved by the local ethics committee in Stockholm County (application #2009/1112-31).

### Treatment

All patients suffered from severe TBI (GCS 3–8 at admission) and were intubated, mechanically ventilated, and sedated with morphine, midazolam, or propofol. If mass lesions were present, they were evacuated as deemed appropriate. Intracranial pressure (ICP) was measured using an extra ventricular drain (EVD) (Medtronic, USA). The transducer for the EVD was placed at the level of the foramen of Monroe. Patients’ heads were elevated to a 30° angle. If traumatic subarachnoid hemorrhage was substantial, transcranial doppler was performed and Nimodipine treatment given. Initially, clear fluids were provided and within 24 h after trauma, naso-gastric tubing was generally used to constantly feed the patient.

A LiquoGuard^®^ (Möller-Medical, Fulda, Germany) system was used (22, 23) to slowly evacuate CSF. The draining velocity of CSF was set to 2 mL/h. The LiquoGuard^®^ simultaneously measures ICP by monitoring CSF pressure pulsation with the sensor placed at the same height as the EVD transducer (23), making it possible to measure ICP and drain CSF at the same time.

Conventional CSF sampling was performed twice a week as a routine management in patients with EVD to screen for potential infection, or more often if deemed clinically motivated, analyzing CSF-cells (manually counted using microscopy techniques), CSF-lactate, CSF-glucose, CSF-albumin (UniCel DxC 800, Beckman Coulter Inc., Brea, CA, USA), and performing CSF cultures at the Department of Laboratory Medicine, Karolinska University Hospital.

### Admission and biomarker parameters

Glasgow Coma Scale (24) and pupil responsiveness (0), unilateral unresponsiveness (1), or bilateral unresponsiveness (2) were acquired at the admission to the hospital. Injury severity score (ISS) (25) and abbreviated injury score (AIS) were assessed (26). Two biomarkers of brain injury, S100B (27) and neuron-specific enolase (NSE) (28), were analyzed. Serum S100B was sampled every 12 h and peak serum levels of S100B 12–36 h after trauma were acquired as clinical routine (29), using an electrochemiluminescence assay (Elecsys System^®^, Roche Diagnostics, Basel, Switzerland). Peak serum levels of NSE were analyzed using an immunoradiometric assays (LIAISON^®^, DiaSorin, Italy) (30).

### Neuroradiology

Intracranial lesions at admission were noted and graded according to Marshall CT-score (31), Rotterdam CT-Score (32), and Stockholm CT-Score (33). The patients were assessed regarding catheter placement according to a previous definition (13, 34); pericontusional location (within 2 cm of a mass lesion, contusion, or hematoma border) or ipsilateral (further away than 2 cm of any lesion, yet in the affected hemisphere), as seen on the postoperative CT scan.

### Outcome

A physician, board certified in neuro-rehabilitation, examined the patients 6 months after trauma assessing extended Glasgow Outcome Score (GOSe) (35). GOSe is graded in eight levels where GOSe 1 = death and GOSe 8 = upper good recovery. GOSe have been previously dichotomized into unfavorable (GOSe 1–4) vs favorable (GOSe 5–8) outcome (36), and GOSe 1–6 vs GOSe 7–8 (15). In the current study, outcome was dichotomized as unfavorable (GOSe 1–5) and favorable (GOSe 6–8), as this also correlated in our study with conventional Glasgow Outcome Score (GOS) dichotomized into unfavorable (GOS 1–3) and favorable (GOS 4–5) outcome.

### Microdialysis

**Figure 1:**
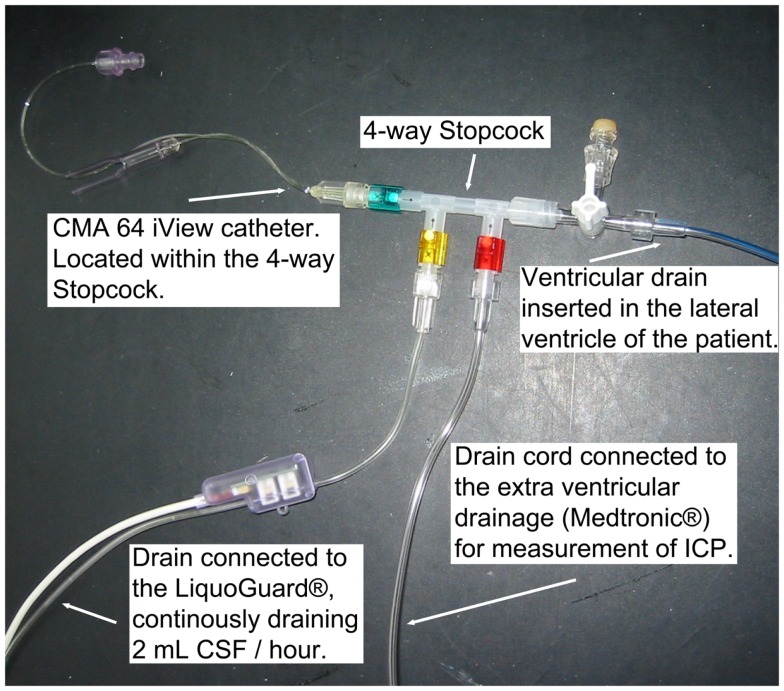
**The monitoring setup, illustrating the CMA 64 MD catheter in a closed system of flowing CSF**.

A 20 kDa cutoff cerebral MD catheter (CMA 70, 10 mm membrane, μ-dialysis AB, Stockholm, Sweden) was inserted into the brain parenchyma, adjacent to the ventricular drain in the affected hemisphere in a diffuse brain injury or when further craniotomy was not performed, or next to the lesion in a focal brain injury, during the initial neurosurgery at admission. Post-surgery at the NICU, the MD catheter was connected to a MD pump (CMA 106, μ-dialysis AB, Stockholm, Sweden) where a commercially available perfusion fluid (“Perfusion Fluid CNS”, μ-dialysis AB, Stockholm, Sweden), pumped at 0.3 μL/min, was used as carrier for all MD metabolites. Another 20 kDa cut off catheter (CMA 64 iView, 10 mm membrane, μ-dialysis AB, Stockholm, Sweden), with a CMA 106 MD pump, was placed inside a four-way stopcock (Multiflo 3, BD Connecta, Franklin Lakes, NJ, USA) (Figure 1), connected to the LiquoGuard^®^ CSF-pump, located in the draining CSF at all times. Membrane length and dialysis perfusion flow rate are factors known to affect recovery and were thus standardized for all catheters used (37). The CMA64 catheter has, when placed in a peripheral vein, shown an adequate congruence (80% MD recovery) between CMA64 MD-glucose and plasma glucose levels (38, 39).

The MD pumps acquired samples were stored in microvials (holding 200 μL). The microvials were analyzed simultaneously every hour with a CMA 600 enzyme photometric analyzer (μ-dialysis AB, Stockholm, Sweden). The CMA 70 catheter has been shown to have a 65–72% substance recovery from the ECF (40). Withdrawal of MD monitoring was performed if the patient became conscious or the catheter was accidentally removed or malfunctioned in any other way.

Median data, and interquartile range, of all MD parameters (glycerol data not normally distributed) were used in the analysis, as in previous studies (11, 15).

The latest MD-CSF sample acquired was compared to the conventional CSF sample that was drawn, as not to affect the local concentrations in the flowing CSF measured by MD-CSF. Recovery was assessed by calculating the MD/CSF ratio for glucose, as well as lactate, for each CSF sample obtained and compared to the closest acquired MD-sample.

### Statistical analysis

The statistical program R (R Foundation for Statistical Computing, Vienna, Austria; http://www.R-project.org) was used. The “rms”-package in R was used to perform univariate logistic regression analyses, analyzing the correlations between differences of CSF and MD-CSF glucose and MD-CSF lactate, as well as for outcome predictions. As a consequence of the limited sample size, a Bonferroni correction (×9) was used to decrease the rate of a potential false positive result (41). Bland–Altman plots were used to visualize congruence between conventional CSF samples and MD-CSF samples (42), adjusted for repeated measures (43). A Mann–Whitney *U* Test was used to assess association between median MD parameters and favorable and unfavorable outcome. Trend curves displaying the different MD metabolites were illustrated with linear plots built in Graph Pad Prism 6.0 (GraphPad Software Inc., 2014).

## Results

### Epidemiological data

The admission characteristics of the 14 patients included are described in Table 1. A total of six patients had a favorable outcome (GOSe 6–8), while eight had an unfavorable outcome (GOSe 1–5), among them, two patients died. Catheter placement was predominantly non-pericontusional (*n* = 9).

**Table 1:** **Demographics**.

Patient	Admission parameters						Biomarkers		CT-classification			Outcome		MD catheter
	Gender	Age	GCS	ISS	AISS	Pupils	S100B	NSE	Marshall	Rotterdam	Stockholm	NICU stay	GOSe	Localization
1	M	54	7	29	4	1	0.26	15	VI	5	3.9	6	7	Pericontusional
2	F	53	5	25	5	2	0.7	73	VI	6	3.8	24	4	Ipsilateral
3	M	23	7	29	4	1	0.33	62	VI	5	2.7	18	5	Pericontusional
4	M	20	8	17	3	0	0.2	27	II	3	1.9	21	8	Ipsilateral
5	M	38	5	38	5	0	0.19	55	II	3	3.0	19	5	Ipsilateral
6	M	25	7	25	5	0	0.26	27	VI	4	2.0	8	8	Ipsilateral
7	M	42	3	38	5	0	0.37	20	II	3	2.5	23	4	Pericontusional
8	F	52	3	29	4	1	0.41	39	II	2	4.0	22	3	Ipsilateral
9	M	59	5	25	5	1	0.62	64	VI	5	2.7	15	2	Pericontusional
10	M	62	7	25	5	0	0.61	31	VI	3	2.0	23	1	Pericontusional
11	M	49	3	16	4	0	0.23	21	II	3	1.7	21	7	Ipsilateral
12	M	20	5	29	4	0	0.12	20	VI	2	2.0	7	8	Ipsilateral
13	F	61	3	26	5	0	0.29	24	VI	5	2.5	4	6	Ipsilateral
14	M	47	4	26	5	0	7.0	149	VI	6	3.5	7	1	Ipsilateral

*Table illustrating patient demographic data. The patients with pericontusional microdialysis catheters are in white rows, while the rows of patients with ipsilateral microdialysis catheters are gray*.

*GCS, Glasgow Coma Scale at admission (3–15). ISS, Injury severity score at admission to the neuro-intensive care unit (1–75). AISS, Abbreviated injury score (1–6). Pupils: 0, Normal responsiveness. 1, unilateral pupil unresponsiveness. 2, bilateral pupil unresponsiveness. S100B, Peak serum levels (μg/L) 12–36 h after reported trauma. NSE, Peak serum levels of NSE (μg/L) during the first 48 h after reported trauma*.

CT-classification: Admission CT scan according to:

Marshall, According to Marshall CT-classification;

*Diffuse injury I – No visible intracranial pathology seen on CT scan*.

*Diffuse injury II – Cisterns are present with midline shift of 0–5 mm and/or lesions densities present; no high or mixed density lesion >25 cm^3^. May include bone fragments and foreign bodies*.

*Diffuse injury III – Cisterns compressed or absent with midline shift of 0–5 mm; no high or mixed density lesion >25 cm^3^*.

*Diffuse injury IV – Midline shift >5 mm; no high or mixed density lesion >25 cm^3^*.

Evacuated mass lesion (Grade V) – Any lesion surgically evacuated

*Non-evacuated mass lesion (Grade VI) – High or mixed density lesion >25 cm^3^; not surgically evacuated*.

*Rotterdam = According to Rotterdam CT-classification score (1–6)*.

*Stockholm = According to Stockholm CT-score (tally)*.

*NICU stay, Days spent in the NICU. GOSe, Extended Glasgow Outcome Score 6 months after trauma (1–8). MD-catheter localization: pericontusional ≤2 cm to the injury; Ipsilateral >2 cm from the injury, but in the affected hemisphere*.

Table 2 shows all the acquired MD data as median levels (and 1st–3rd quartiles). The number of MD samples acquired for each patient varied between *n* = 30 and *n* = 265 per patient, depending on NICU stay and sample type. Thus, Table 2 also provides the duration of MD monitoring in hours from admission, 1 day up to about 11 days. Table 3 illustrates the conventional CSF samples and the different parameters analyzed.

**Table 2:** **Microdialysis parameters**.

Patient	Brain-MD					CSF-MD				
	Glucose (mmol/L)	Lactate (mmol/L)	Pyruvate (μmol/L)	LPR	Glycerol (μmol/L)	Glucose (mmol/L)	Lactate (mmol/L)	Pyruvate (μmol/L)	LPR	Glycerol (μmol/L)
1	1.9 (0.9–3.2) *n* = 45	5.9 (3.1–8.5) *n* = 45	141 (93–224) *n* = 44	49 (34–54) *n* = 45	106 (89–125) *n* = 45	4.7 (2.4–5.0) *n* = 31	2.0 (1.9–2.2) *n* = 32	73 (62–83) *n* = 30	27 (24–33) *n* = 31	57 (27–62) *n* = 31
2	1.4 (0.6–1.8) *n* = 157	4.9 (4.1–6.2) *n* = 154	148 (135–168) *n* = 156	33 (30–35) *n* = 154	181 (143–310) *n* = 157	5.0 (4.8–5.4) *n* = 113	2.9 (2.5–3.4) *n* = 112	85 (75–100) *n* = 105	34 (28–42) *n* = 105	73 (44–103) *n* = 114
3	0.6 (0.5–0.8) *n* = 163	3.5 (2.8–4.2) *n* = 160	100 (72–144) *n* = 149	35 (29–43) *n* = 148	361 (242–874) *n* = 158	4.9 (4.3–5.5) *n* = 110	2.4 (2.2–2.7) *n* = 108	113 (105–123) *n* = 94	22 (19–25) *n* = 93	32 (22–40) *n* = 111
4	1.6 (1.1–2.1) *n* = 241	2.6 (2.3–3.1) *n* = 263	112 (88–126) *n* = 265	24 (22–29) *n* = 262	987 (706–1420) *n* = 259	5.5 (4.4–6.9) *n* = 125	1.8 (1.6–2.1) *n* = 124	82 (67–99) *n* = 124	22 (20–25) *n* = 124	38 (32–51) *n* = 125
5	4.0 (3.2–4.5) *n* = 129	2.5 (2.0–3.2) *n* = 124	130 (112–147) *n* = 122	19 (16–25) *n* = 118	483 (243–877) *n* = 127	4.8 (4.5–5.1) *n* = 125	2.3 (1.9–2.5) *n* = 123	94 (80–111) *n* = 122	23 (18–29) *n* = 116	99 (86–112) *n* = 125
6	5.9 (5.5–6.3) *n* = 55	2.1 (1.9–2.4) *n* = 58	130 (122–149) *n* = 50	15 (15–18) *n* = 46	87 (77–107) *n* = 58	4.8 (4.6–5.1) *n* = 97	1.7 (1.6–1.9) *n* = 96	63 (60–69) *n* = 96	27 (24–29) *n* = 92	29 (25–34) *n* = 96
7	1.6 (1.1–2.2) *n* = 112	7.4 (6.2–8.6) *n* = 119	269 (255–284) *n* = 118	27 (23–32) *n* = 125	120 (66–238) *n* = 118	5.1 (4.6–5.5) *n* = 110	3.2 (2.9–3.4) *n* = 116	124 (108–158) *n* = 114	25 (20–31) *n* = 119	44 (39–57) *n* = 115
8	0.5 (0.3–1.1) *n* = 75	7.2 (4.9–7.8) *n* = 67	157 (128–185) *n* = 68	45 (40–49) *n* = 64	184 (91–359) *n* = 70	4.3 (3.9–4.8) *n* = 147	2.7 (2.5–2.9) *n* = 149	112 (101–124) *n* = 146	24 (23–27) *n* = 146	64 (55–76) *n* = 148
9	1.2 (0.8–1.5) *n* = 213	5.0 (3.6–6.3) *n* = 230	137 (92–173) *n* = 216	35 (31–40) *n* = 215	87 (58–115) *n* = 215	5.2 (4.6–6.0) *n* = 150	3.6 (3.2–4.1) *n* = 151	151 (138–172) *n* = 148	24 (22–26) *n* = 145	58 (38–72) *n* = 149
10	1.0 (0.8–1.3) *n* = 89	2.6 (2.2–3.0) *n* = 93	102 (94–117) *n* = 89	24 (22–26) *n* = 89	2273 (1817–2529) *n* = 94	5.6 (4.7–7.0) *n* = 123	4.7 (4.1–5.1) *n* = 128	199 (179–228) *n* = 125	23 (20–26) *n* = 125	82 (66–113) *n* = 127
11	0.9 (0.8–1.2) *n* = 151	3.4 (2.9–3.9) *n* = 152	112 (92–125) *n* = 148	32 (29–34) *n* = 145	451 (269–1133) *n* = 153	5.4 (4.9–5.9) *n* = 150	2.8 (2.6–3.1) *n* = 145	100 (94–114) *n* = 142	28 (25–30) *n* = 139	66 (58–75) *n* = 147
12	1.0 (0.7–1.2) *n* = 100	5.2 (4.5–5.8) *n* = 99	166 (154–183) *n* = 99	31 (28–34) *n* = 99	114 (71–149) *n* = 102	4.7 (4.2–5.1) *n* = 106	2.3 (2.0–2.7) *n* = 106	77 (72–89) *n* = 104	29 (26–34) *n* = 104	40 (37–46) *n* = 107
13	0.2 (0.1–1.1) *n* = 42	17.1 (15.3–18.8) *n* = 62	133 (114–270) *n* = 58	129 (49–143) *n* = 62	133 (118–153) *n* = 55	5.6 (5.3–5.8 *n* = 50	2.4 (2.2–2.6) *n* = 50	126 (99–149) *n* = 50	20 (17–23) *n* = 50	53 (46–62) *n* = 49
14	1.8 (1.5–2.4) *n* = 54	14.9 (13.8–16.2) *n* = 54	447 (365–528) *n* = 54	33 (31–37) *n* = 58	605 (495–892) *n* = 54	4.3 (3.3–5.2) *n* = 51	4.8 (3.3–5.8) *n* = 51	239 (194–267) *n* = 49	19 (18–23) *n* = 53	49 (38–71) *n* = 50

*Brain-MD and CSF-MD parameters in median values, with 1st–3rd quartile in parenthesis. Sample size is the amount of MD samples acquired of the specific metabolite for each patient. The patients with pericontusional microdialysis catheters are in white rows, while the rows of patients with ipsilateral microdialysis catheters are gray. The amount of samples represents total monitored time (h)*.

**Table 3:** **CSF samples**.

	Median (±1st–3rd quartile)
Samples per patient	2.0 (1.25–2.75)
Day(s) from MD surgery to CSF sampling	3 (2–5) (days)
CSF-Glucose	5.1 (4.8–5.8) (mmol/L)
CSF-Lactate	2.6 (2.1–3.2) (mmol/L)
CSF-Erythrocytes	5200 (1000–38,400) (mm^3^)
CSF-Leukocytes	25 (8–89) (mm^3^)
CSF-Albumin	140 (36–283) (mg/L)
Time between MD sample and CSF sample	25 (16–44) (min)
MD recovery – glucose	0.98 (0.90–1.03), if median per patient 0.96 (0.86–1.00)
MD recovery – lactate	0.97 (0.84–1.08), if median per patient 0.98 (0.83–1.08)

*Table of the 29 cerebrospinal fluid (CSF) samples acquired. Presented as average and standard deviation (SD), or median ±1st–3rd quartile, depending on sample distribution. Samples per patient, days from microdialysis (MD) surgery sampling, CSF-glucose (mmol/L), CSF-lactate (mmol/L), CSF-erythrocytes (red blood cell count, RBC, per mm^3^), CSF-leukocytes (white blood cell count, WBC, per mm^3^), CSF-albumin (mg/L), time between MD sample and CSF sample (min, MD always prior to CSF sample), MD recovery – glucose (MD-CSF glucose/CSF-glucose), and MD recovery – lactate (MD-CSF lactate/CSF-lactate) all presented as continuous variables. The MD recovery is presented as both per pair sample and as averaged (median) pair samples per patient*.

### Correlation between CSF and MD-CSF samples

**Figure 2:**
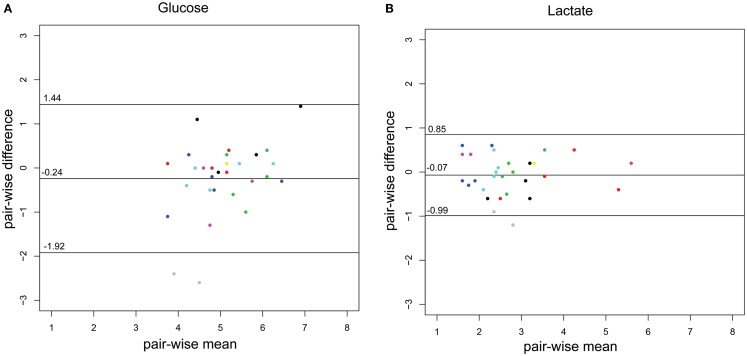
**Bland–Altman plots of the CSF-glucose and CSF-lactate samples (*n* = 29) corresponding with MD-CSF glucose (A) and MD-CSF lactate (B)**. The points are plotted with the difference between two observations (pair-wise difference) on the *y*-axis, and the mean of the two observations (mean-wise difference) on the *x*-axis. The confidence limits and the mean are plotted as black lines. The variance for glucose and lactate are 0.51 and 0.13, respectively. Every patient (*n* = 14) is represented by a unique color. Two points lay outside the confidence limits in **(A)** (glucose) while one point is outside the confidence limits in **(B)** (lactate).

The concordance of the two methods is illustrated using a Bland–Altman plot (Figures 2A,B), adapted for repeated measures, where the variance for glucose and lactate are 0.51 and 0.13, respectively.

As is illustrated in Table 3, the median MD recovery (the pair-wise CSF-MD sample divided by CSF sample) of glucose and lactate was 0.98 (interquartile range: 0.90–1.03) and 0.97 (0.84–1.08) (if averaged per patient, 0.96 and 0.98), respectively. The difference between MD-CSF samples and CSF samples were not significantly related to by CSF-erythrocytes, CSF-leukocytes, CSF-albumin, or the time from insertion of the catheter to sampling (Table 4). CSF-lactate levels correlated significantly to CSF-erythrocyte levels (*p* = 0.0035, *r*^2^ = 0.255). None of the patients developed positive CSF cultures during their NICU stay.

**Table 4:** **Influence of different parameters on the difference (Δ, delta) between glucose and lactate in CSF-MD and CSF, respectively**.

Parameters	Delta glucose (CSF-MD – CSF-glucose) *p*-Value	Delta lactate (CSF-MD– CSF-lactate) *p*-Value
Time from insertion of MD catheter	0.0941	0.8446
CSF-Erytrocyte	0.5244	0.0780
CSF-Leukocytes	0.6837	0.2037
CSF-Albumin	0.8902	0.2691
Time from MD and CSF sample	0.6857	0.2547

*Influence of different parameters on the delta level between CSF-MD and conventional CSF samples using a univariate regression analysis. None of the analyzed parameters yielded statistical significant results*.

### Correlation between MD parameters and outcome

**Figure 3:**
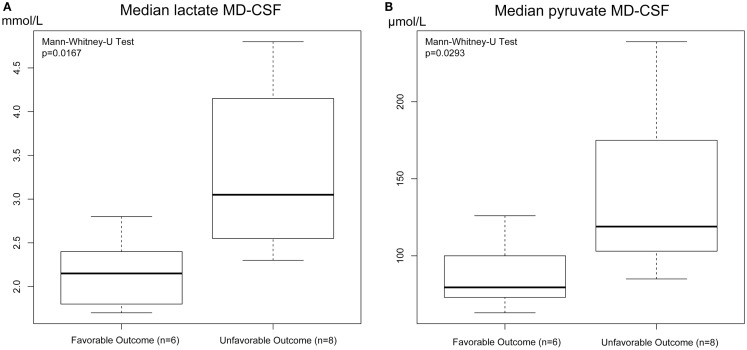
**Box plot of median MD-CSF lactate (A) and MD-CSF pyruvate (B) levels presented in relation to outcome (GOSe 1–5 vs 6–8)**. MD-CSF lactate levels and MD-CSF pyruvate levels are significantly higher in patients with unfavorable outcome (*p* = 0.0167 and *p* = 0.0293, respectively, Mann–Whitney *U* Test.

Both MD-CSF lactate (*p* = 0.0167) and pyruvate (*p* = 0.0293) levels were significantly lower in the favorable outcome (GOSe 6–8) group compared to the unfavorable group (GOSe 1–5) (Figures 3A,B). The regional MD-Brain did not show any significant difference in outcome (Table 5).

**Table 5:** **Parameters and univariate correlation to patient outcome**.

Parameters	*p*-Value	Pseudo-*r*^2^	*p*-Value after Bonferroni correction
**BRAIN-MD**			
Glucose	0.2799	NS	NA
Lactate	0.1041	NS	NA
Pyruvate	0.2512	NS	NA
Glycerol	0.6364	NS	NA
Lactate:pyruvate ratio	0.3555	NS	NA
**CSF-MD**			
Glucose	0.8234	NS	NA
**Lactate**	**0.0057**	**0.578**	0.0513
**Pyruvate**	**0.0011**	**0.732**	**0.0099**
Glycerol	0.1167	NS	NA
Lactate:pyruvate ratio	0.1783	NS	NA
CSF-parameter
**Erytrocytes**	**0.0003**	**0.821**	**0.0027**

*MD parameters from each patient (*n* = 14) in Brain-MD and CSF-MD (median levels) correlated to patient outcome (GOSe 1–5 vs GOSe 6–8) using univariate regression analysis. A Bonferroni correction was used to improve accuracy of the model. Higher levels of lactate, pyruvate and erythrocytes in the CSF correlate to worse outcome. NS, not significant. NA, not applicable. Bold indicates a significant *p*-value*.

### MD-CSF and MD-Brain monitoring of patients

**Figure 4:**
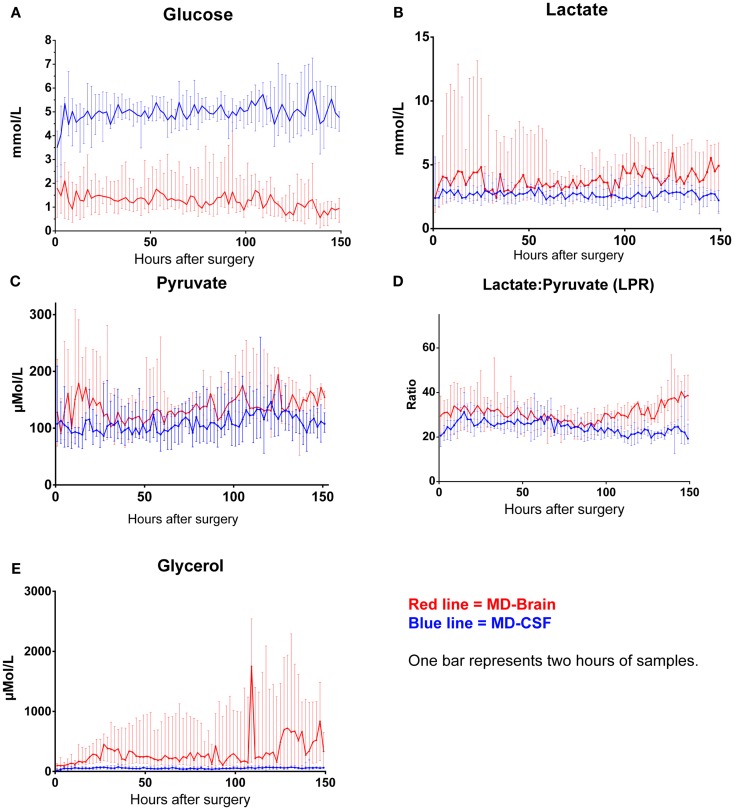
**Median (± 1st–3rd quartile) CSF-MD and Brain-MD glucose (A), lactate (B), pyruvate (C), LPR (D), and glycerol (E) levels for all patients (*n* = 14)**.

**Figure 5:**
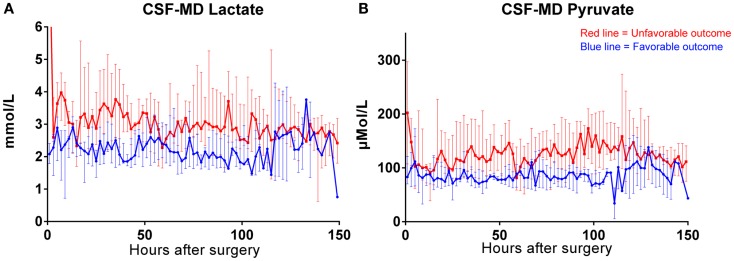
**Median (± 1st–3rd quartile) CSF-MD lactate (A) and pyruvate (B) levels in patients with favorable (*n* = 6) (GOSe 6–8) and unfavorable (GOSe 1–5) outcome (*n* = 8)**. One bar represents two samples.

The levels of MD-CSF and MD-Brain in the first 150 h after insertion are displayed in Figures 4A–E. Glucose is higher in the MD-CSF compared to MD-Brain, while the opposite applies for lactate, pyruvate, LPR, and glycerol that are generally higher in MD-Brain compared to MD-CSF. Figures 5A,B illustrates, what is also seen in Figures 3A,B, that patients with favorable outcome have lower levels of lactate and pyruvate in MD-CSF compared to patients with unfavorable outcome (Figures 5A,B).

## Discussion

The comparison of “global,” CSF microdialysis, conventional CSF samples, and intracerebral microdialysis has, to the best of our knowledge, never been studied before in a clinical setting. This study of 14 patients indicates that the samples acquired using the current “global” CSF-MD method is highly correlated to conventionally drawn CSF samples concerning glucose and lactate and that the median MD recovery of the 20 kD CMA64 MD catheter with a 0.3 μL/min dialysis flow rate in CSF is near 100%, higher than previously described for 20 kD MD catheters in ECF (40). In addition, MD-CSF pyruvate and lactate levels were, despite this small cohort, significantly correlated to outcome.

### CSF parameters and MD-CSF parameters

The difference between CSF and MD-CSF parameters is illustrated by the Bland–Altman plots (Figures 2A,B). Lactate levels are deemed to be within an acceptable range for clinical use, with only one sample outside the confidence limits. The variance for glucose was higher (0.51) than for lactate (0.13). For glucose, several samples were inside the confidence bounds, yet 32% of the samples were outside 1 SD (±0.84 mmol/L), which could represent a problem if the method was to be clinically implemented. As can be seen for glucose and lactate, one patient (gray dots) significantly lowered the confidence bounds. Also, for glucose, another patient (black) had higher pair-wise difference. These patients could represent that the catheter is in some way malfunctioning and subsequently affect the sampling and the Bland–Altman plot.

Leukocyte, albumin, and erythrocyte concentrations in CSF, as well as the time from MD catheter insertion and sampling, are parameters that could affect the function and recovery of the MD catheter. However, no significant relation was found between these parameters and the differences between MD-CSF and conventional CSF levels of glucose and lactate (Table 4).

Glucose levels exhibited a higher variance than lactate, perhaps because of a higher fluctuation of the metabolite in CSF. Further studies would be needed to elucidate if this is a recurrent observation.

### MD recovery

The amount of substance extracted from a known concentration using MD technique has previously been described as a “recovery” or a “relative recovery” (40, 44).

The median MD recoveries of glucose and lactate were 0.98 and 0.97, respectively. Previous data have shown (*n* = 3) that the mean “relative recovery,” using a CMA70 MD-Brain catheter in brain parenchyma, perfused with 0.3 μL/min ringer solution, in an extrapolation-to-zero-flow model, was 0.65 for glucose and 0.67 for lactate (40). In our model, the correct concentration in CSF was known, hence no extrapolation was necessary. However, it could be difficult to compare these MD recoveries due to the different models used, different MD catheters (CMA64 vs CMA70) used and conditions between CSF and ECF.

The catheters both have the same diameter, membrane size, and other membrane specific properties, and differ only in the material that was used to make the plastic shaft (polyamid for the CMA70 and polyamid ether sulfone for the CMA64) (personal communication, μ-Dialysis AB, Stockholm, Sweden), making the choice of catheter an unlikely reason for the discrepancy.

Jacobson et al. suggested that the concentration of the metabolites just outside the MD catheter is lower in ECF due to local depletion (45), which may not be applicable in CSF, as the CSF has a higher circulation and is continuously being renewed. Dahlin et al. were able to increase the MD recovery by using human CSF as perfusion fluid in an *in vitro* model (46). The MD recovery for the CMA64 catheter in peripheral blood is about 80% for glucose (39), lower than what is seen in our study.

In our system, metabolites from a flow of CSF were measured using a CMA64 MD catheter, which might be the cause of the higher observed MD recovery if compared to the studies by Hutchinson et al. (65–67%) (40) and Rooyackers et al. (80%) (39). This finding was in coherence with the manufacturer, who routinely checks the calibration of MD catheters and measures levels of metabolites in known concentrations, *in vitro*. They have found that the MD recovery is close to 100% if the catheter is placed in a water-like fluid (personal communication, μ-Dialysis AB, Stockholm, Sweden). Their findings were similar to our own; in order to improve the recovery for the MD catheter, it is probable that the analyzed media, in themselves, are more important than the perfusion velocity and MD catheter membrane characteristics.

In conclusion, the MD recovery of glucose and lactate were higher than previously described, perhaps explained by the different conditions between ECF and CSF or dissimilarity with previously described methods.

### Glucose

The levels of glucose in MD-CSF remained higher than MD-Brain throughout the first 150 h of monitoring (Figure 4A). There was no significant correlation to outcome for neither MD-CSF glucose nor MD-Brain glucose. The levels of MD-Brain glucose were similar to other studies analyzing glucose in ECF (47), while ECF levels from uninjured patients have been shown to be 0.6–2.6 mmol/L (48).

Glucose is transported directly from the blood to the ECF through the blood–brain barrier using transporter proteins (primarily GLUT1), and ECF levels are considered more stable than plasma levels due to the transporter proteins ability to adapt to hyper/hypoglycemic conditions (49). The level of glucose in the ECF is lower than the CSF due to the increased metabolism in the brain parenchyma. While the levels of glucose between ECF and serum have been studied in both healthy patients (50) and TBI patients (51), and shown to be around 40% of the serum level (52), little is studied about the movement of glucose between the ECF and CSF. Hochwald et al. postulated that glucose enters the CSF from the blood using facilitated transport (53) and that the transport follows saturable kinetics as the extraction rate from serum is independent of the CSF-glucose concentration (54). Second, the flow of glucose between CSF and ECF has been shown to occur via oubain-sensitive (and oubain-insensitive) fluxes and diffusion, with imminent changes between the two compartments (55).

The flow of labeled glucose between CSF and ECF could be easily analyzed using the current setup, making it suitable for further research utilizing the MD technique.

### Lactate and pyruvate

The levels of lactate were generally lower in MD-CSF compared to the brain ECF (MD-brain) (Table 2; Figure 4B). Normal CSF levels of lactate and pyruvate have been shown to be around 1.01–2.09 mmol/L and 30–150 μmol/L, respectively, not being significantly affected by gender (56), while ECF levels in uninjured patients are 2.0–2.9 mmol/L and 120–166 μmol/L (lactate and pyruvate, respectively) (48).

Isolated lactate increase in CSF after TBI has been shown in several studies (57, 58), where it has been correlated to patient outcome and a subsequent increase of ICP (58). Guerra-Romero et al. intrathecally injected rabbits (*n* = 3) with sodium lactate. The animals were monitored with MD in the ECF, and showed (59) a subsequent increase of CSF-lactate, while no significant changes could be detected in the brain ECF, hence probably indicating a regulated metabolic control of lactate between the two compartments. Increased metabolism leads to higher levels of lactate in the brain parenchyma, which may subsequently be measured in the MD-Brain catheter. The range of lactate levels detected were higher in MD-Brain compared to MD-CSF, and as seen in the study by Guerra-Romero, an increase of lactate in one of the cerebral compartments may not lead to an increase in the other.

Increased MD-CSF lactate and pyruvate levels were correlated to an unfavorable outcome (Table 5; Figures 5A,B). No other MD parameters were significantly correlated to outcome. Also, a *post hoc* analysis revealed that the significance for MD-CSF lactate and pyruvate remained when different dichotomizations of GOSe were used (15, 36). However, the LPR, commonly used as a marker for ischemia when monitoring with brain-MD, and previously shown to correlate to unfavorable outcome (11, 60), was not elevated. Increased lactate and pyruvate levels in the CSF, following TBI (61, 62) and subarachnoid hemorrhage (63), without any effect on the LPR, have been seen in previous studies (61, 63). This has been suggested to be an effect of red blood cell glycolysis in the CSF and brain parenchyma (63). This is supported by a study from 1969 where blood was injected into the CSF of cats (*n* = 6), with a subsequent increase of both lactate and pyruvate levels. The authors suggested that an increased glycolysis of the CSF/blood cell mixture was the reason to this metabolic pattern (64). Using MD-Brain, a similar pattern has been shown where lactate and pyruvate levels increase following secondary brain hypoxia in human TBI patients (65). In this study, several patients with increased ischemia (PbtO_2_ below 10 mmHg) had an increase of pyruvate levels, concurrent with increased lactate levels, leaving the LPR unchanged. The authors postulate that this increase of pyruvate represents glycolysis exceeding the ability of the ischemic-affected brain tissue to adequately metabolize pyruvate (65).

In the present study, the erythrocyte concentration in CSF correlated significantly to the lactate levels in CSF, and to patient outcome (Table 5), which we believe to be the most probable cause of this metabolic pattern of increased lactate and pyruvate levels in CSF. The severity of subarachnoid, and intraventricular hemorrhage after TBI has been extensively correlated to patient outcome (66), which could explain why these patients had a worse outcome.

Other possible mechanisms for the increased lactate and pyruvate could be hypermetabolism, a common problem following TBI (67). Regional seizure activity in the brain might be another reason (68), even though clinical representation of epileptic seizure were treated at our NICU with antiepileptic medication and intermittently monitored with EEG, some non-convulsive seizure activity could be missed.

As is seen in Table 1, the MD-Brain catheters were inserted in pericontusional tissue in 36% of the cases (*n* = 5), while the other (*n* = 9) where placed further from the affected brain parenchyma. These catheters would be expected to show higher levels of glycerol and LPR, as well as lower glucose, but no difference was found (Table 2). A recent study proposes that a more global approach to MD monitoring is beneficial (15), with catheters placed in healthy tissue in order to determine the presence of cerebral metabolic crisis (increased lactate and LPR levels, and decreased glucose levels).

In aggregate, the metabolic pattern of increased lactate and pyruvate levels in CSF found in our study is believed to be the result of high levels of erythrocytes in the CSF, yet other metabolic causes in the affected brain cannot be excluded.

### Glycerol

Levels of MD-Brain glycerol were higher than MD-CSF (Table 2; Figure 4E). Normal ECF levels of glycerol have been reported to be around 20–80 μmol/L (48) and 8.6–25 μmol/L in CSF (69). The glycerol levels in our study were higher in both ECF and CSF. ECF and CSF levels of glycerol in the intact brain are both related to serum levels of glycerol (70). Increased levels probably correlate to ongoing cell death, due to its abundance in cell membranes (7). It has been postulated by Nau et al. that prolonged persistence of glycerol in the CSF may reflect similar conditions in the ECF (69).

Perhaps due to the relatively short half-life of glycerol in CSF (1.03–3.68 h) and the high flow rate of the CSF, there is a concentration difference between ECF and CSF (69), with an ongoing release in the injured brain, hence increased levels of glycerol in the ECF compared to the CSF.

### The current setup for monitoring patients with meningitis

The current MD-CSF technique enhances the temporal resolution when monitoring CSF parameters in NICU patients, sampling lactate, and glucose levels every hour. An ICP-guided therapy for patients suffering from acute BM has been shown to improve outcome, compared to traditional therapy (71), and the current monitoring setup, where levels of CSF metabolites may be readily accessible, would definitely assist in this type of neuro-intensive care treatment.

### Limitations

The limited sample size provides several obvious limitations to this study. However, the method has analyzed a total of *n* = 7448 MD-CSF samples and *n* = 8358 MD-Brain samples generating accurate data for the median levels for the *n* = 14 patients that could be included. To avoid intra-patient data (dependent and independent data) to affect the analysis, the Bland–Altman plots were adjusted for repeated measures causing wider confidence intervals (Figures 2A,B). The analyses in Table 4 would in theory require a mixed models approach due to repeated measures, this is not possible as several patients only have one data point and all data have been treated as independent data points.

Only 5 of the 14 Brain-MD catheters ended up in the pericontusional locale, an area that has been suggested to better represent tissue at risk after TBI (8). This heterogeneity could explain why Brain-MD samples did not correlate to outcome as well as CSF-MD samples did, even if MD monitoring of “uninjured” brain tissue also has been shown to be a good marker for global metabolic crisis and to be correlated to outcome (15).

The timing of samples when comparing MD-CSF and conventional CSF lagged up to about one hour, since the conventional CSF sampling was not performed at any regular time during the day. However, Table 4 reveals that there was no significant difference between the MD-CSF and conventional CSF levels of glucose and lactate, in regard of the timing when they were acquired.

In fact, all the pumps used in this study cause a sampling delay from the actual monitored metabolic event that could be significant. This is, however, also a limitation using the MD technique, but it is amplified when also using pumped CSF, as it is hard to determine exactly when the potential harmful biochemical event occurred when MD-CSF samples are to be temporally compared to MD-Brain samples.

## Conclusion

This new technique of global MD-CSF monitoring correlates with conventional CSF levels of glucose and lactate. The MD recovery, using the current MD set up in CSF, is close to 100% for both lactate and glucose. Increase in lactate and pyruvate, without any effect on the LPR, significantly correlates to unfavorable outcome, though perhaps indicating an effect of the presence of erythrocytes in the CSF, or possibly a hypermetabolic state in the injured brain. Additional studies, including increased sample sizes, are necessary to further validate the method and the current findings.

## Conflict of Interest Statement

The authors declare that the research was conducted in the absence of any commercial or financial relationships that could be construed as a potential conflict of interest.
